# Effects of bromocriptine mesylate on homocysteine and high-sensitivity C-reactive protein levels in patients with type-2 diabetes mellitus

**DOI:** 10.15171/jcvtr.2016.02

**Published:** 2016-03-14

**Authors:** Mitra Niafar, Leili Pourafkari, Kavus Shahsavarinia, Nushin Milanchian, Farhad Niafar, Nader D. Nader

**Affiliations:** ^1^Endocrine Research Center, Tabriz University of Medical Sciences, Tabriz, Iran; ^2^Department of Anesthesiology, University at Buffalo, Buffalo, NY, USA

**Keywords:** Homocysteine, Bromocriptine, CRP

## Abstract

***Introduction:*** Quick release bromocriptine (BROM-QR), currently approved for glycemic control, reduces the risk of cardiovascular events in adults with type-2 diabetes mellitus (T2DM). This study evaluates the effect of BROM-QR on homocysteine (HOMC) and high sensitive C-reactive protein (hs-CRP), the biochemical markers of coronary atherosclerosis/inflammation, in patients with uncontrolled T2DM.

***Methods:*** In this non-randomized, before-and-after clinical trial, patients with uncontrolled T2DM on stable doses of two oral hypoglycemic agents received BROM-QR for 6 months. The change in serum concentrations of HOMC was the primary endpoint. Anthropometric measurements such as body mass index (BMI) and waist circumference were measured at the baseline and at the completion of treatment along with fasting plasma glucose (FPG), HbA1c, total cholesterol, triglyceride, creatinine and hs-CRP. Multivariate regression analysis was performed to identify factors associated with changes in the levels of HOMC.

***Results:*** In 64 patients (46 completed 6 months of treatment), age was 55±7 years and the duration of T2DM was 8.0 ± 4.4 years. On enrollment, mean HbA1c, FPG, hs-CRP and HOMC levels were 9.0± 1.3 percent, 184 ± 42 mg/dL, 3.8± 3.4 mg/dl and 10.8 ± 6.2 micromole/L; respectively. Mean decrease of 0.7 ± 1.1 percent for HbA1c (*P* = 0.001) and 22 ± 44 mg/dL for FPG was observed (*P* = 0.002). HOMC levels decreased to 8.5 ± 5.2 micromole/L (*P* = 0.011) while hs-CRP levels remained unchanged at 3.7 ± 2.9 mg/dL (*P* = 0.835).

***Conclusion:*** While HOMC and HbA1c levels decreased significantly after 6 months of treatment with BROM-QR in patients with T2DM, serum levels of hs-CRP, total cholesterol and triglyceride did not significantly change.

## Introduction

Bromocriptine mesylate quick release (BROM-QR) is approved for glycemic control in patients with type-2 diabetes mellitus (T2DM). This drug is a centrally acting dopamine receptor agonist that resets the circadian peak of dopaminergic signal. Although the exact mechanisms are yet to be elucidated, BROM-QR exerts its unique insulin-sensitizing effect through setting off a shift in the caloric intake.^1,2^ A recently published meta-analysis reports that BROM-QR reduces HbA1c and fasting blood sugar (FBS) without affecting the lipid profile or body mass index (BMI).^3^ BROM-QR is further shown to reduce adverse cardiovascular events^4^ and to decrease the risk myocardial reinfarction and cardiovascular mortality.^5^ Homocysteine (HOMC) is derived from an essential amino acid methionine that is believed to contribute to vascular injury and is generally considered as a marker for nephropathy and retinopathy among diabetics.^6-9^ In patients with T2DM, higher levels of HOMC are independently associated with increased risk of cardiovascular events.^10^ C-reactive protein, a classical acute-phase reactant, is a liver-derived protein that increases in inflammatory states.^11^ High-sensitivity C-reactive protein (hs-CRP) is elevated in diabetics and is an independent predictor of increased cardiovascular risk.^11,12^ Antidiabetic agents with insulin-sensitizing properties like glitazone and metfromin have been shown to reduce hs-CRP levels.^13^ On the other hand, oral hypoglycemic agents that increase insulin secretion such as sulphonylureas are not found to significantly alter concentrations of hs-CRP.^14^ A 6-month treatment with BROM-QR had no significant effect on HOMC or hs-CRP levels when prescribed for patients with elevated prolactin levels.^15^ Potential effects of BROM-QR on HOMC and hs-CRP levels in T2DM have not been investigated. We aimed to examine the effects of a 6-month adjuvant BROM-QR therapy in patients with uncontrolled T2DM on metformin and sulphonylurea. Primary outcome variables were changes in HOMC levels. Secondary outcome variables were changes in hs-CRP, HbA1C and BMI.

## Patients and methods

### Study design and setting

This study was designed as a before and after clinical trial and it was registered under trial No. 201204156710N3 in the clinical trial registry (http://www.IRCT.ir). The Research Ethics Committee of Tabriz University of Medical Sciences reviewed and approved the study protocol and the informed consent form.

### Inclusion and exclusion criteria

All T2DM patients who were poorly controlled (glycosylated hemoglobin, HbA1c > 7.5%^16^) on two oral hypoglycemic drugs (metformin 2 g per day and glibenclamide 15 mg per day) were screened and enrolled into the study after signing an informed consent. Inclusion criteria consisted of a diagnosis of T2DM and receiving stable doses of metformin and sulphonylurea for the past 3 months with a recent HbA1c more than 7.5%. Exclusion criteria included patient refusal, age less than 30 years and more than 65 years, insulin therapy, pregnancy, lactation, clinically significant comorbid conditions, history of syncope, psychosis, allergy to ergot-related or BROM-QR drugs. Additionally, patients with stage ≥4 of chronic kidney disease (CKD) with glomerular filtration rate less than 30 ml/min, and those receiving vitamin B6, vitamin B12 or acid folic supplements were excluded.

### Study protocol

After enrollment and obtaining demographic, anthropometric measurement and relevant clinical information, patients were prescribed BROM-QR at the dose of 1.25 mg/day. Dose was increased by 1.25 mg daily every three days until a stable dose of 2.5 mg twice a day was reached if the patients could tolerate without developing significant gastrointestinal symptoms. Anthropometric measurements included height (cm), weight (kg), waist circumference (cm) and calculation of BMI during their first clinic appointment. FBS (mg/dL), postprandial blood glucose (mg/dL), HbA1c percentage, total cholesterol (mg/dL), triglycerides (mg/dL), high-density lipoproteins (HDL, mg/dL), low-density lipoproteins (LDL, mg/dL) and serum creatinine (mg/dl) were measured. Additionally, a comprehensive medical history consisting full physical examination and complete medication reconciliation was obtained during the first clinic visit. Follow-up visits were performed at 1.5, 3 for assessing the quality of glycemic control and medication compliance any possible drug-related complication; however, the data obtained from their final study visit 6 months after the date of enrollment and treatment. From 64 patients, 46 completed the full course of the treatment and remained in the study. FBS, postprandial blood glucose and HbA1c were measured at 3 months and 6 months. Lipid profile, hs-CRP and HOMC concentrations were measured at 6 months follow-up.

### Sample size and power analysis

Since no previous data was available regarding the effects of BROM-QR on HOMC levels, we used the data on 20 patients as the pilot phase. The serum concentrations of HOMC were 9.92±7.32 mg/dL at the baseline, which decreased to 6.21±3.87 mg/dL after 6 months of BROM-QR therapy (the overall standard deviation was 5.5 mg/dL). A power analysis was performed using the online sample size calculator of the University of British Columbia (http://www.ubc.edu/stat). A total of 35 patients were required for a power of 0.80 and alpha error of 0.05. With 46 patients who completed the follow-up period and were compliant with 6-month BROM-QR treatment, the power of the study was 0.90.

### Statistical analysis

SPSS version 22.0 (IBM^®^, Chicago, IL) was used for data analysis. Changes in binary variables after the intervention were assessed using McNemar test and were reported as frequencies with the percentage of the total number of the patients prior and after treatment. Continuous variables were examined by paired *t* tests and were reported as mean ± standard deviation (SD) if they had normal distribution pattern. The study variables were expressed as mean ± SD with 95% CI. Null hypotheses were rejected if *P* values were less than 0.05.

## Results

### Baseline characteristics

**Figure 1:**
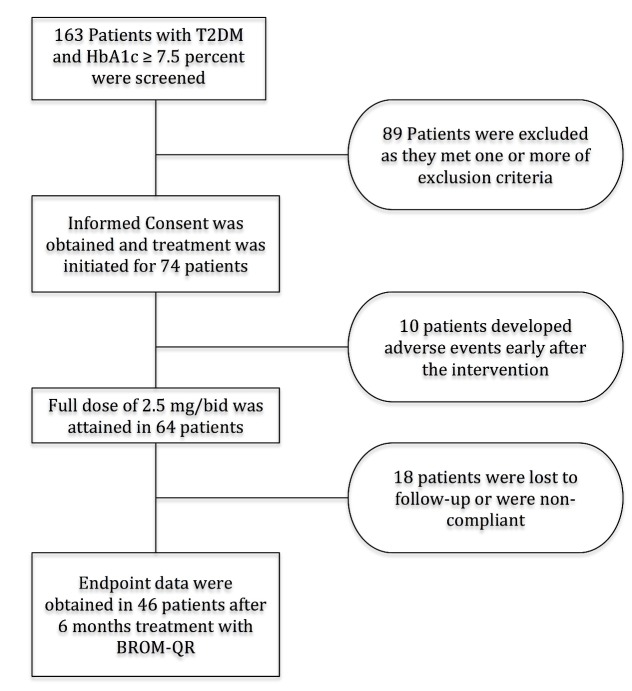
Flowchart diagram depicting the inclusion process.

One hundred and sixty three patients with uncontrolled T2DM were screened. Eighty-nine patients were excluded because they met one or more of the exclusion criteria for enrollment. Seventy-four eligible patients signed informed consent prior to the initiation of treatment. Ten patients discontinued treatment because of the side effects consisting dizziness, nausea and headache. In 64 patients (45 females), the dose BROM-QR was maximized to 5 mg per day (Figure 1). Mean age of patients was 55.0 ± 7.2 years and the mean duration of the disease was 8.0 ± 4.4 years. The enrolled patients were in average 1.60 ± 0.09 meters tall and there were 2 patients in <25 kg/m^2^, 32 patients in 25-30 kg/m^2^, and 30 patients in ≥30 kg/m^2^ BMI categories. There was no morbidly obese patient, as the calculated BMI did not exceed 35 kg/m^2^ in any cases. Baseline characteristics of the study group are provided in Table 1.

**Table 1:** Demographic distribution of the patient population at the time of enrollment and conclusion of the study

**Variables**	**At the time of enrollment ** **N=64**	**At the conclusion of the study ** **N=46**
Female/ Male	45/19	29/17
Age (y)	55.0 ± 7.2	54.5 ± 7.6
Duration of diabetes (y)	8.0 ± 4.4	8.0 ± 4.4
Height (m)	1.60 ± 0.09	1.60 ± 0.09
Weight (kg)	78.5 ± 13.5	78.7 ± 13.2
Body mass index (kg/m^2^)	30.9 ± 4.5	30.6 ± 4.2
Waist circumference (cm)	108 ± 9	106 ± 9
Systolic BP (mm Hg)	127 ± 19	127 ± 16
Diastolic BP (mm Hg)	78 ± 9	76 ± 7

Abbreviation: BP, blood pressure.

### Post-treatment data

A total of 46 patients were compliant to the treatment protocol and were available to follow up at the conclusion of 6 months. At the conclusion of the study 47.8% achieved HbA1c ≤7.5% and had controlled diabetes. However, in 13.0% HbA1c was between 7.5% to 8%, and in 39.1% HbA1c was higher than 8%. All paired comparisons were done exclusively in patients who completed 6-month treatment period. There was no significant difference in* s*ystolic or diastolic blood pressure (+1.9±6.4 mm Hg in mean arterial blood pressure; *P*=0.258). There was a significant increase in weight by +0.7±2.5 kg (*P*=0.021) while FBS, postprandial blood glucose and HbA1c significantly decreased at 6-months follow-up (*P* values of 0.002, 0.001 and 0.001; respectively). There were no significant changes in serum concentrations of total cholesterol, triglycerides, LDL or HDL among patients following BROM-QR treatment (Table 2).

**Table 2:** Metabolic laboratory values and renal function are shown upon enrollment and after treatment for the patients who completed BROM-QR treatment protocol for 6 months

	Pre-treatment	Post-treatment	Treatment changes [95% CI]	P-Value
Fasting blood sugar (mg/dL)	184 ± 48	165 ± 47	↓-19 [-33–(-6)]	0.007
Postprandial glucose (mg/dL)	281 ± 71	205 ± 65	↓-76 [-99–(-53)]	<0.001
Hemoglobin A1C (% gram)	8.9 ± 1.5	8.1 ± 1.5	↓-0.8 [-1.1–(-0.4)]	<0.001
Total cholesterol (mg/dL)	177 ± 44	169 ± 35	-8 [-22–6]	0.263
Triglycerides (mg/dL)	188 ± 88	172 ± 65	-16 [-39–7]	0.320
HDL (mg/dL)	45.6 ± 9.6	46.8 ± 10	+1.2 [-1.5–4.0]	0.174
LDL (mg/dL)	96 ± 35	87 ± 31	-9 [-20–3]	0.138
Serum creatinine (mg/dL)	0.95 ± 0.22	0.90 ± 0.15	-0.05 [-0.090–0.003]	0.065
Glomerular filtration rate (mL/min)	83.9 ± 23.4	87.7 ± 20.0	↑+3.8 [0.49–2.32]	0.026

**Figure 2:**
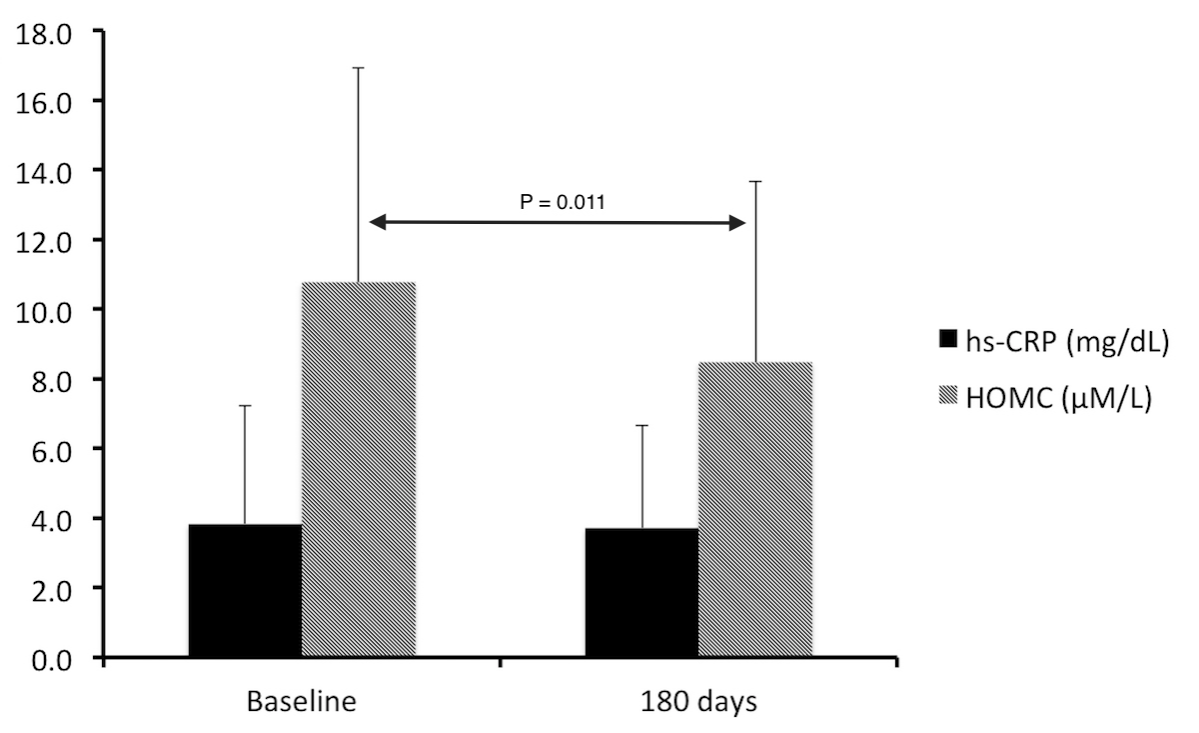
Levels of homocysteine (HOMC) and high sensitivity C-reactive protein (hs-CRP) at baseline and 180 days after treatment.

Interestingly, serum creatinine concentration had a strong trend toward decreasing (-0.04±0.02 mg/dL; *P*=0.065) as the estimated glomerular filtration rate significantly increased (+3.8±1.7 mL/min; *P*=0.026) following BROM-QR therapy. Mean hs-CRP level was 3.8±3.4 mg/dl upon enrollment that remained basically unchanged at 3.7 ± 2.9 mg/dl at the conclusion of the treatment plan (*P*=0.835). Serum concentrations of HOMC were 10.8 ± 6.2 µM/L and significantly reduced to 8.5 ± 5.2 µM/L after 6 months of treatment with BROM-QR (*P*=0.011). Figure 2 illustrates the observed changes in the levels of these two inflammatory markers during the study.

## Discussion

In the present study, 6 months of a BROM-QR treatment in patients with T2DM expectedly lowered FBS, postprandial glucose and HbA1c. Moreover, HOMC levels also significantly decreased, though hs-CRP levels failed to change significantly. T2DM treatment has been evolving with new class of drugs being introduced during the past 2 decades. Bromocriptine had been used for years in the treatment of acromegaly, Parkinson disease and hyperprolactinemia.^1^ The quick release formulation of this drug was approved in 2009 as an adjunct in the treatment of T2DM.^17^ A few studies have addressed the effects of adjunct bromocriptine on cardiovascular events.^4,5,18^

CYCLOSET trial enrolled more than 3000 patients in a 52-week, randomized, double-blind multicenter trial the frequency of serious adverse were comparable between the study arms.^18^ A stricter look at the same population evaluating only the cardiovascular outcomes, endorsed the original observation and confirmed the reduction in the major adverse cardiovascular events in the treatment group.^4^ In a randomized, double blind study on the subjects of CYCLOSET trial, on the impact of 12- month treatment with BROM-QR over cardiovascular disease; treatment reduced the CVD endpoints by 52% in the on-treatment group in patients with good glycemic control and HbA1c <7%.^5^

Though the exact mechanism of action is not fully elucidated for bromocriptine, modulation of circadian hypothalamic function and the resultant reduction in the sympathetic nervous system activity have been forwarded as the plausible candidates. Increased sympathetic output leads to vascular injury through increased inflammatory cytokines, increased production of free fatty acids and its direct effects over the vasculature.^19-21^

Elevated serum levels of HOMC as a non-traditional marker of atherosclerosis associates with alterations of endothelial and smooth muscle cell functions.^22,23^ While lowering the serum levels of HOMC can be efficiently and inexpensively achieved by folic acid and vitamin B supplementation,^24,25^ studies have failed to show any cardiovascular benefit from lowering HOMC levels other than possibly a reduction in stroke rate.^26^ Thus routine screen and treatment for increased levels is not recommended.^27^ Nevertheless it should be kept in mind that some of these studies were performed in subjects consuming folate fortified food and had a relatively short follow-up.^28^

HOMC levels are increased in CKD. Though the mechanism is not clarified, decreased clearance of HOMC in these patients is the likely cause.^29^ Moreover, in a study enrolling patients with GFR between 20 and 55 mL/min/1.73 m^2^ increased HOMC was identified as a risk factor for incident cardiovascular events,^30^ in another study on patients with stage 3 or 4 CKD the relationship between mortality was lost after adjustment for kidney function.^31^ In a randomized controlled trial 6-month treatment with BROM-QR in 28 patients with T2DM, a decrease in blood pressure and left ventricular mass was reported, while creatinine clearance remained unchanged.^32^

We observed a small but significant increase in GFR along with a decrease in HOMC in our patients. Whether anti-inflammatory effects of BROM-QR cause the decrease in HOMC level or it is simply a reflection of increased glomerular filtration needs further evaluation. A few studies have suggested possible anti-inflammatory effects of bromocriptine.^33,34^ Since there has not been any decrease in hs-CRP in our patient population, the renal excretion of HOMC seems to be the likely mechanism for reduction of this amino acid in the serum.

## Limitations

Measuring the other inflammatory markers such as tumor necrosis factor alpha or interlukin-6 would have added to the inflammatory hypothesis of this intervention. Without such measurements the proposed hypothetical mechanism is merely a speculation. Additionally, for the evaluation of potential protective effects of BROM-QR on kidney function, the finding of improvement in glomerular filtration was an unexpected finding for us, and therefore a quantitative evaluation of proteinuria particular excretion of HOMC in the urine and its specific clearance was not included in our original protocol.

## Ethical issues

The Research Ethics Committee of Tabriz University of Medical Sciences reviewed and approved the study protocol and the informed consent form.

## Competing interests

Authors declare no conflict of interest in this study.
